# Gonioscopy‐Assisted Transluminal Trabeculotomy in Angle Recession Glaucoma: A Case Report

**DOI:** 10.1155/crop/4075105

**Published:** 2026-04-29

**Authors:** Abdullah Bin Hemid, Basel M. Alsolami

**Affiliations:** ^1^ Ophthalmology, King Abdullah Medical City, Makkah, Saudi Arabia, kamc.med.sa

**Keywords:** angle recession glaucoma, gonioscopy-assisted transluminal trabeculotomy (GATT), traumatic glaucoma

## Abstract

**Introduction:**

We report a case of angle recession glaucoma (ARG) in a 15‐year‐old male following blunt ocular trauma, managed with gonioscopy‐assisted transluminal trabeculotomy (GATT).

**Case Presentation:**

Despite maximal medical therapy, intraocular pressure (IOP) remained uncontrolled. Gonioscopy revealed approximately 5 clock hours of superior angle recession. The patient underwent GATT, which resulted in an immediate postoperative reduction in IOP, complicated by transient hyphema. At 1 year follow‐up, the patient achieved stable IOP (12 mmHg) without anti‐glaucoma medications, full visual recovery (20/20), and complete resolution of hyphema.

**Conclusion:**

This case highlights the potential role of GATT as an effective, minimally invasive surgical option for managing ARG.

## 1. Introduction

ARG is a type of secondary open‐angle glaucoma that develops following blunt ocular trauma. It was first described by Collins in 1892 as a traumatic tear between the circular and longitudinal muscle fibers of the ciliary body [1]. Early IOP elevation in ARG may result from trabecular meshwork (TM) injury, Schlemm’s canal (SC) dysfunction, inflammation, or hyphema. In contrast, late‐onset IOP elevation is associated with structural changes, including fibrosis of the TM and SC or the formation of a hyaline membrane over the TM [2–4].

Management of ARG includes both medical and surgical options [5]. Conventional surgical interventions, such as trabeculectomy and glaucoma drainage device (GDD) implantation, are widely used; however, their long‐term outcomes remain variable [6–8].

This case report highlights the potential role of GATT in the management of ARG.

## 2. Case Report

A 15‐year‐old medically fit male was referred to our center, complaining of decreased vision and redness in the right eye for the past few days, preceded by ocular trauma with a ball while playing football 2 weeks prior to presentation.

The patient’s general examination and vital signs were within normal limits, with no external injuries to the head or face. Ocular examination revealed visual acuity of 20/20 in both eyes, with IOP of 28 mmHg in the right eye and 16 mmHg in the left eye, despite the patient being on maximal anti‐glaucoma therapy, including topical medications and oral acetazolamide 500 mg twice daily.

**Figure 1:**
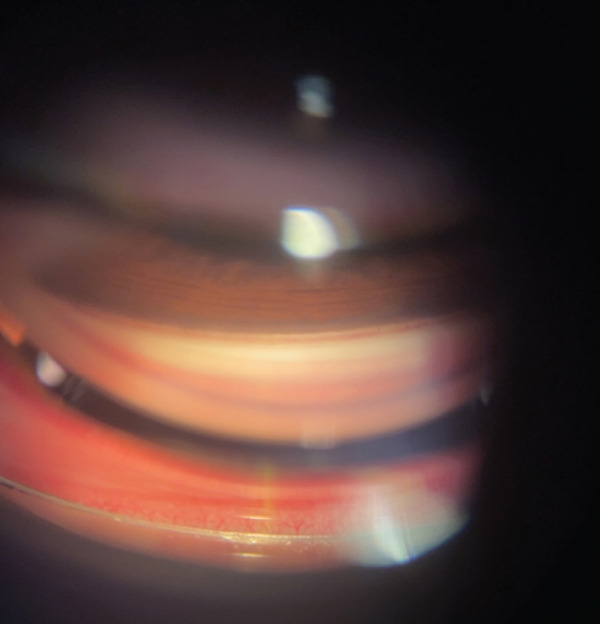
Angle recession of approximately 5 clock hours superiorly of the right eye.

Pupil examination showed a fixed, mid‐dilated pupil in the right eye, while the left pupil was round, regular, and reactive. Extraocular movements were normal in all gazes. Slit‐lamp examination of the right eye showed mild conjunctival ciliary injection, a clear cornea, a deep and quiet anterior chamber (AC), a clear lens, and clear vitreous. Fundus examination revealed a cup‐to‐disc ratio of 0.6, a good foveal reflex, and a flat retina 360° with no retinal tears. The left eye examination was unremarkable. Gonioscopy revealed Grade 4 open angles in both eyes, with approximately 5 clock hours of superior angle recession in the right eye (Figure 1), while the left eye showed no angle recession.

A diagnosis of ARG in the right eye was established. Because of uncontrolled IOP despite maximal medical therapy, the decision was made to proceed with GATT after discussion with the patient and family.

The procedure was performed under sterile conditions. A 1‐mm paracentesis was created using a microvitreoretinal (MVR) blade, and viscoelastic material was injected into the AC. A 2.2‐mm temporal clear corneal incision was made. A 5‐0 Prolene suture was cauterized to form a mushroom tip and introduced into Schlemm’s canal after incising the TM. The suture was advanced circumferentially for 360°, then withdrawn to complete the trabeculotomy. Approximately 50% of the viscoelastic and an air bubble were left in the AC, and the wound was confirmed to be watertight.

On postoperative day one, visual acuity in the right eye was hand motion, with an IOP of 11 mmHg. Examination revealed a clear cornea, a sealed incision, an air bubble, dispersed hyphema, and an inferotemporal clotted hyphema. The patient was started on prednisolone acetate 1% every 2 h, moxifloxacin 0.5% every 6 h, brimonidine 0.15% twice daily, and a combination of antibiotics (neomycin and polymyxin B) with a corticosteroid (dexamethasone 0.1%) ointment at bedtime. One week postoperatively, vision improved to 20/20 with an IOP of 7 mmHg. Examination showed a small residual inferior clotted hyphema (< 0.1 mm).

At one‐month, visual acuity remained 20/20 with an IOP of 10 mmHg. A persistent inferior clotted hyphema (0.2 mm) was observed. The patient underwent endoscopic cyclophotocoagulation (ECP) to identify a potential bleeding source. No active bleeding was identified; however, a suspicious area at the 6 o’clock position was cauterized.

At 6 months postoperatively, IOP remained well controlled at 12 mmHg without anti‐glaucoma medications, and the hyphema had completely resolved. At the most recent visit, 1 year postoperatively, vision remained stable, with IOP maintained at 12 mmHg without treatment.

## 3. Discussion

Angle recession is a clinical finding seen in patients with a history of non‐penetrating ocular trauma. It results from a tear between the longitudinal and circular muscle fibers of the ciliary body, often involving the TM. IOP elevation in ARG may develop months to years following the initial injury [9]. It is estimated that 1%–20% of eyes with angle recession progress to glaucoma [1].

Multiple mechanisms contribute to the development of ARG. Blunt trauma compresses the globe axially and expands it equatorially. Rapid corneal indentation forces aqueous humor laterally, deepening the AC and increasing the diameter of the corneoscleral limbal ring, which damages the AC angle. Injury to the ciliary body leaves the longitudinal ciliary muscle fibers attached to the scleral spur, while the circular muscle, pars plicata, and iris become detached and displaced posteriorly. This shearing injury may damage branches of the anterior ciliary arteries, leading to hyphema [10, 11].

Early IOP elevation may result from direct damage to the TM and SC. Over time, fibrosis and scarring of these structures can lead to persistent elevation of IOP. Additional proposed mechanisms include reduced tension of the ciliary muscle on the scleral spur, leading to narrowing of SC, as well as the formation of a hyaline membrane over the TM [12].

Initial management typically involves medical therapy; however, many cases fail to achieve adequate IOP control with medications alone. Surgical intervention is indicated in cases of persistent IOP elevation despite maximal medical therapy [13].

Laser trabeculoplasty has shown limited efficacy in ARG. Data from the American Academy of Ophthalmology IRIS Registry report a 47.5% failure rate at 18 months. Argon laser trabeculoplasty (ALT) has not demonstrated sustained IOP reduction, and selective laser trabeculoplasty (SLT), although not extensively studied, is generally considered ineffective [14]. Nevertheless, Al‐Obaida et al. reported a 20% reduction in IOP with SLT in a small case series, warranting further investigation [15].

ARG has been associated with an increased risk of trabeculectomy failure. Reported success rates are lower in ARG compared with primary open‐angle glaucoma (43% vs. 74%). Similarly, outcomes of GDD implantation in ARG appear inferior to those of trabeculectomy without antimetabolites [16, 17].

Minimally invasive glaucoma surgeries (MIGS) offer several advantages, including a favorable safety profile, lower complication rates, and preservation of conjunctival integrity, allowing for future filtering procedures if needed [18, 19]. However, the optimal MIGS procedure for ARG remains unclear. Given that ARG is primarily characterized by impaired aqueous outflow due to TM dysfunction, GATT may provide a targeted approach by circumferentially bypassing the dysfunctional TM. This enables direct access of aqueous humor to the collector channels, aqueous veins, and the episcleral venous system.

Recent studies suggest a potential role of GATT in the management of ARG. Elubous et al. reported effective IOP reduction with sustained control at 1 year in a patient with ARG [20]. Similarly, Mirza et al. demonstrated a significant IOP reduction at one‐year follow‐up in a small case series, which was associated with reduced medication use, further supporting the efficacy of GATT as a targeted surgical approach for trabecular outflow dysfunction [21].

## 4. Conclusion

This report contributes to the limited literature on the surgical management of ARG and highlights the potential role of GATT as an effective treatment option. Further research is needed to evaluate its long‐term efficacy and outcomes.

## Funding

No funding or grant support was received.

## Disclosure

All authors meet the current ICMJE authorship criteria.

## Consent

Written consent to publish this case was not obtained. This report does not contain any identifiable personal information.

## Conflicts of Interest

The authors declare no conflicts of interest.

## Data Availability

The data that support the findings of this study are available from the corresponding author upon reasonable request.
